# Recent advances in understanding *Propionibacterium acnes *( *Cutibacterium acnes*) in acne

**DOI:** 10.12688/f1000research.15659.1

**Published:** 2018-12-19

**Authors:** Eftychia Platsidaki, Clio Dessinioti

**Affiliations:** 1 Department of Dermatology, Andreas Syggros Hospital, University of Athens, Athens, Greece

**Keywords:** Priopionibacterium acnes, biofilm, phylotypes, acne, antimicrobial resistance

## Abstract

The skin commensal *Propionibacterium acnes*, recently renamed *Cutibacterium acnes*, along with the other major pathophysiological factors of increased seborrhea, hyperkeratinization of the pilosebaceous unit, and inflammation, has long been implicated in the pathogenesis of acne. Recent advances have contributed to our understanding of the role of *P. acnes* in acne. Although there are no quantitative differences in *P. acnes* of the skin of patients with acne compared with controls, the *P. acnes* phylogenic groups display distinct genetic and phenotypic characteristics, *P. acnes* biofilms are more frequent in acne, and different phylotypes may induce distinct immune responses in acne. *P. acnes* plays a further important role in the homeostasis of the skin’s microbiome, interacting with other cutaneous commensal or pathogenic microorganisms such as *Staphylococcus epidermidis*, *Streptococcus pyogenes*, and *Pseudomonas *species. In the era of increasing antimicrobial resistance, the selection of acne treatment targeting *P. acnes* and the prevention of antibiotic resistance play a key role in improving outcomes in acne patients and public health.

## Introduction

The cutaneous microbiome exists in a finely tuned equilibrium in healthy skin that when perturbed may lead to various inflammatory skin diseases. The three most commonly observed cutaneous genera are *Corynebacteria*, *Propionibacteria*, and *Staphylococci* ^1^.

*Propionibacterium acnes* has been implicated in the pathophysiology of prostate cancer ^2^, sarcoidosis ^3^, infective endocarditis ^4^, infections involving prosthetic devices (such as prosthetic joints, central nervous system ventricular shunts, and cardiac implantable devices) ^5^, and acne, the last of which is the focus of this review. *P. acnes* is a Gram-positive, non-spore-forming human skin commensal that prefers anaerobic growth conditions ^6, 7^. It is a member of the normal skin microbiota along with *P. avidum*, *P. granulosum*, and *P. humerusii* ^8^. The *P. acnes* genome is 2.5 Mb in size and has been completely sequenced. It has genes encoding metabolic enzymes, enabling it to survive in microaerophilic conditions, but also lipases that degrade the lipids of the pilosebaceous follicle, providing the bacterium with the energy it needs ^9^. Recently, a taxonomic reclassification was proposed in which *P. acnes* was renamed *Cutibacterium acnes* to account for genomic adaptive changes and to differentiate it from other *Propionibacteria* species. In particular, specific lipase genes were identified encoding for triacylglycerol lipase and lysophospholipase able to degrade sebum lipids ^10^. However, it has been proposed that it is taxonomically valid to continue to use the genus name *Propionibacterium* for the cutaneous group within dermatology specialties for a range of different reasons, including to avoid confusion with the previous name, *Corynebacterium acnes* ^11^.

In this review, we describe the characteristics of *P. acnes* concerning taxonomy, the role of different phylotypes and *P. acnes* biofilm in acne pathophysiology, and the targeting of *P. acnes* with appropriate acne treatments and the respective implications in the homeostasis of the skin’s microbiome and the emergence of antimicrobial resistance.

## *P. acnes* taxonomy

With regard to taxonomy, *P. acnes* has been classified into three phylotypes (phylogroups) based on gene sequences or biological characteristics (lipase activity): I, II, and III ^12^. These phylogroups in turn have been split into distinct subspecies known as *P. acnes* subsp. *acnes*, *P. acnes* subsp. *defenden*s, and *P. acnes* subsp. *elongatum*, respectively, to denote distinct phylogenetic, genomic, and phenotypic characteristics as well as their association with different clinical diseases, including acne and progressive macular hypomelanosis ^13, 14^.

The subspecies *P. acnes* subsp. *acnes* has been described. Extracellular enzymes include RNase, neuraminidase, hyaluronidase, acid phosphatase, lecithinase, and lipase. The bacterial cells ferment glucose, and lactic acid is produced from fermentable carbohydrates in variable quantities. The major long-chain fatty acid produced is 13-methyltetradecanoic acid ^14^. Gene sequence analysis of *P. acnes* on the basis of the genes *recA* and *tly* revealed further phylogenetic subdivisions within the type I clade: the types IA, IB, and IC. Higher-resolution methods provided additional differentiation of IA strains into types IA1 and IA2 ^13^. So *P. acnes* is subdivided into six phylotypes: IA1, IA2, IB, IC, II, and III ^15^. Multi-locus sequence typing and single-locus sequence typing (SLST) identified further subgroups among phylotypes, called clonal complexes.

The *P. acnes* phylogroups have been associated with specific diseases and distinct virulence, biochemical, and immunological characteristics that will be discussed in the following section.

## *P. acnes* in acne: the role of distinct phylotypes

*P. acnes* has been regarded as an important member of the cutaneous microbiota. It has been linked to the inflammatory skin condition acne vulgaris for more than 100 years. The four major pathophysiological factors implicated in the pathogenesis of acne include the role of *P. acnes*, increased seborrhea, hyperkeratinization of the pilosebaceous unit, and inflammation ^16^.

*P. acnes* colonization of the skin is necessary but not sufficient for the establishment of acne pathology. *P. acnes* dominates the microbiota of pilosebaceous units and accounts for 87% of clones in patients with acne and in individuals without acne ^17^. *P. acnes* has been reported to represent more than 30% of the facial microbiota in patients with acne ^1^, but another study of 55 patients with facial acne reported lower rates (less than 2%) of sampled bacteria ^18^. These results should be interpreted with caution given the role of the sampling methodologies used. Different sampling methods, such as swab, scrape, cyanoacrylate gel biopsy, and needle biopsy, are used to collect skin bacteria for testing. Each technique targets different skin structures and anatomical sites. The sampling of superficial and intra-stratum corneum bacterial populations is considered quite straightforward. However, the sampling of hair follicle populations has proven more difficult and a skin biopsy may be required. The use of tape-stripping for hair follicle sampling in acne can be misleading, as multiple superficial and intra-stratum corneum microbial populations are sampled but bacteria may reside in a deeper part of the hair follicle ^19^. This area is inaccessible with the above-mentioned sampling methodologies, providing very little material from inside the hair follicle and making it difficult to standardize ^8^. *P. acnes* sampling with bacterial culture may not reliably distinguish between *P. acnes* populations with possibly variable pathogenic potential ^20^.

Although there is no quantitative difference of *P. acnes* in the skin of patients with acne compared with controls ^17^, its phylogenic groups display distinct genetic and phenotypic characteristics in acne ^10^ and different ** phylotypes are known to induce distinct immune responses in acne ^12^. Different *P. acnes* types have been isolated from acne vulgaris, and the type III strains have been associated with progressive macular hypomelanosis, underscoring the importance of genetic division of *P. acnes* and suggesting the involvement of specific *P. acnes* phylotypes in the pathophysiology of acne ^21^. ****

Focusing on acne, the typing of *P. acnes* isolates has revealed distinct profiles in patients with acne ( Table 1) ^15, 22, 23^. A case-control study reported loss of *P. acnes* phylotype diversity in patients with severe inflammatory acne, and there was a predominance of phylotype IA1 compared with healthy controls. With additional molecular typing methods, the SLST type A1 was predominant in the acne group ^15^. On the other hand, a small study in 29 patients with mild acne compared with 34 patients with severe acne did not reveal the association of a specific *P. acnes* phylotype with the severity of acne, and phylotype IA1 and SLST type A1 were the predominant types in both groups ^22^.

**Table 1.** The potential role of distinct *Propionibacterium acnes* types in patients with acne.

Study	*P. acnes* phylotypes	SLST types	Acne patients studied	Proposed roles
Dagnelie *et al*. ^15^ (2018)	Predominance of phylotypes IA1 (84.4%) and II	A1	24 patients with severe acne of face and back versus 12 controls	- Decrease of phylotype diversity may be due to hyperseborrhea and qualitative sebum modifications in acne - Loss of diversity may activate innate immunity and trigger inflammatory acne
Nakase *et al*. ^23^ (2017)		- Isolates of clade A (60.3%) predominated - Strains of clade F more frequent in severe acne (40%) compared with mild acne (23.3%) - Phylogenetic type A5 most frequent (29.4%)	113 patients with acne	
Paugam *et al*. ^22^ (2017)	- Phylotype IA1 the most frequent in mild acne (55.2%) and in severe acne (67.6%) - No difference of phylotypes between mild and severe acne groups	A1 predominance with no difference between acne groups	29 patients with mild acne and 34 patients with severe acne	- In a small number of patients, the severity of acne was not associated with a specific *P. acnes* group

SLST, single-locus sequence type.

## The role of the *P. acnes* biofilm

Bacteria may exist as biofilms in their natural habitat. A biofilm is defined as a microbial aggregate embedded in extracellular matrix which protects cells from harmful conditions in the environment and facilitates escaping from host surveillance mechanisms. Burkhart and Burkhart (2007) suggested that *P. acnes* biofilm may penetrate into the sebum and act like an adhesive, leading to the increased cohesiveness of corneocytes and the formation of microcomedones ^24^. Additionally, a high availability of sebum, a nutritional substrate for *P. acnes*, may result in an increased proportion of metabolically active bacteria and contribute to a pro-inflammatory phenotype of the *P. acnes* biofilm. This may explain the acne flares in adolescence, when increased hormone and sebum production are dominant ^25^.

*In vitro* growth of *P. acnes* biofilms demonstrated the composition of the extracellular polymeric substance (EPS) matrix of *P. acnes* biofilm with extracellular DNA, proteins, and glycosyl residues as well as upregulated mRNA expression of Christie–Atkins–Munch-Peterson (CAMP) factor 1 ^26^.

Only one study has investigated *P. acnes* biofilm in acne patients compared with controls. A case-control study in facial biopsies showed that follicular *P. acnes* was more frequently demonstrated in samples from acne patients compared with matching controls. Furthermore, *P. acnes* from acne samples more frequently formed biofilms in the sebaceous follicles compared with control samples ^20^. Although similar biofilms have also been observed in skin diseases other than acne, such as folliculitis, folliculitis decalvans, and hidradenitis suppurativa, these were seen in terminal hair follicles ^27– 29^.

## Target activities of *P. acnes* in acne

**Figure 1.**
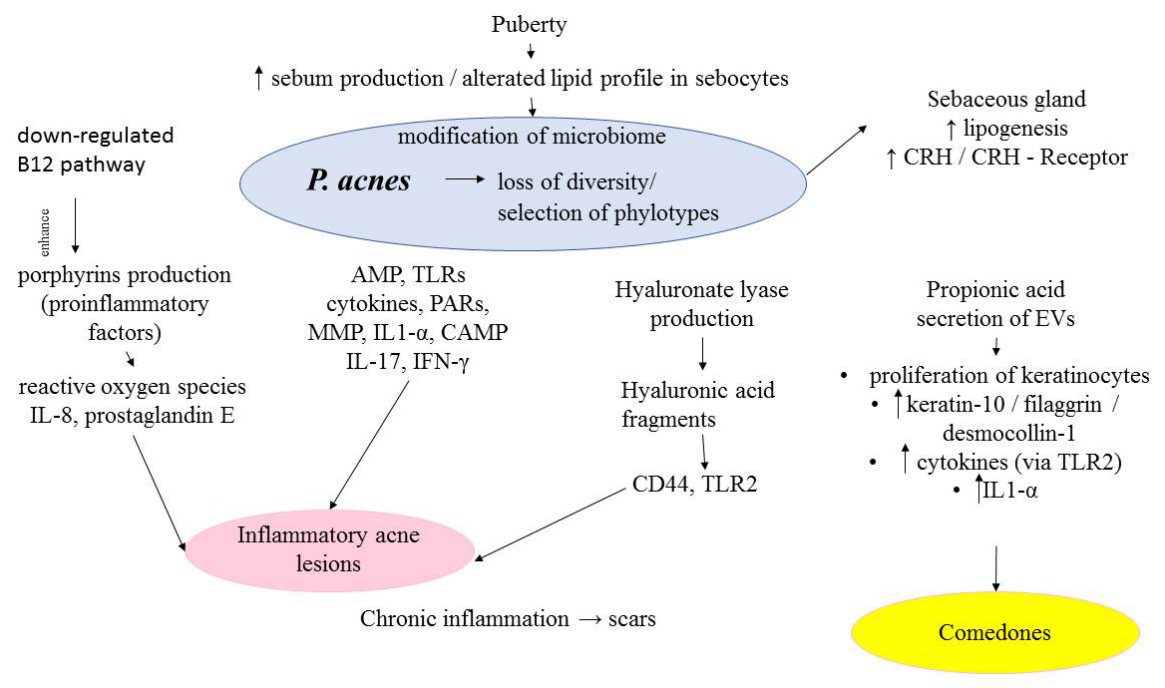
*Propionibacterium acnes*: loss of diversity, selection of phylotypes, and its different target activities in acne. *P. acnes* induces the production of AMP, TLRs, cytokines, PARs, MMP, IL-1a, CAMP, hyaluronate lyase, and porphyrins, resulting in the formation of inflammatory acne lesions. It modulates the differentiation of keratinocytes by inducing keratin 10, filaggrin, and desmocollin 1 expression. It stimulates the sebaceous glands and sebum synthesis via the CRH/CRH receptor pathway. AMP, antimicrobial peptide; CAMP, Christie–Atkins–Munch-Peterson; CRH, corticotropin-releasing hormone; EV, extracellular vesicle; IFNγ, interferon-gamma; IL, interleukin; MMP, matrix metalloproteinase; PAR, protease-activated receptor; TLR, Toll-like receptor.

As *P. acnes* modulates the differentiation of keratinocytes and increases local inflammation, it is regarded as an etiological agent of both the microcomedone (a structure invisible to the naked eye) in the early stages of acne and of the inflammatory acne lesions ^30^. The different target activities of *P. acnes* in acne are summarized in Figure 1.

*P. acnes* shows complex interactions with key events implicated in the pathogenesis of acne. It interacts with the innate immunity, including Toll-like receptors (TLRs), antimicrobial peptides (AMPs), protease-activated receptors (PARs), and matrix metalloproteinase (MMP), and upregulates the secretion of pro-inflammatory cytokines, including interleukin-1a (IL-1a), IL-1β, IL-6, IL-8, IL-12, tumor necrosis factor-alpha (TNF-α), and granulocyte-macrophage colony-stimulating factor (GM-CSF), by human keratinocytes, sebocytes, and macrophages ^16, 31^. Moreover, the production of AMP (LL-37, β-defensin 2), cytokines (IL-1α), and MMP was associated with the increased expression of the G-protein-coupled receptor PAR-2 in keratinocytes from acne-affected skin ^32^. *P. acnes* extracts are directly able to modulate the differentiation of keratinocytes by inducing b1, a3, a6s, aVb6 integrin expression, and filaggrin expression on keratinocytes, changes seen in the development of acne lesions ^33^. Interplay between *P. acnes* and macrophages in the perifollicular dermis can induce IL-1β ^32^, which in turn may further activate the NLRP3-inflammasome pathway in antigen-presenting cells and myeloid cells ^19^. Recent *in vitro* studies have revealed that *P. acnes* can induce IL-17 production by T cells (Th1/Th17) ^31^. Clusters of CD3 ^+^ cells have been demonstrated in the vicinity of the *P. acnes*-positive comedones, cells that were absent from the surrounding inflamed lesions. These findings in early acne stage further support the role of *P. acnes* in the initiation of inflammation ^34^. *P. acnes* releases extracellular vesicles (EVs) which also induce cellular responses via TLR2 signal cascades. These *P. acnes*-derived EVs induce IL-8 and GM-CSF and decrease epidermal keratin-10 and desmocollin, contributing to the development of acne lesions ^35^.

Yu *et al*. showed that acne-associated *P. acnes* phylotypes induced distinct cytokine patterns *in vitro* in peripheral blood mononuclear cells from healthy individuals, including higher levels of inflammatory interferon-gamma (IFN-γ) and IL-17, suggesting a mechanism of inducing acne via both Th1 and Th17 pathways ^12^. On the other hand, *P. acnes* phylotypes associated with healthy skin induced higher levels of IL-10. Moreover, there were different expression patterns between phylotypes; acne-associated phylotypes showed higher expression of an adhesion protein, whereas phylotypes associated with healthy skin showed higher expression of a cell surface hydrolase. These identified immune responses and proteomes of different *P. acnes* strains provided deeper insight into how specific *P. acnes* phylotypes influence the pathogenesis of acne ^12^. In a follow-up study, Agak *et al*. reported differential effects of acne-affected skin- and healthy skin-associated lineages of *P. acnes* on CD4 ^+^ T-cell and Th17 cell responses and suggested that *P. acnes* strains express different antigenic components on their surface structure, possibly explaining the higher IL-17 levels induced in acne-affected skin-associated *P. acnes* strains ^36^.

Furthermore, *P. acnes* has been implicated in lipogenesis and sebum production, as it stimulates the sebaceous glands and sebum synthesis via the corticotropin-releasing hormone (CRH)/CRH receptor pathway ^37^. Expression of the complete CRH system has been described in acne; a study in biopsies from the facial skin of patients with acne reported a stronger expression of CRH in sebocytes of acne-involved skin compared with non-involved and normal skin ^38^. In particular, CRH augments the synthesis of sebaceous lipids and induces IL-6 and IL-8 release by sebocytes, mediated by the CRH receptor ^39^.

A recent study reported that a secretory CAMP factor of *P. acnes* has a role in its cytotoxicity, as mutations of CAMP diminished *P. acnes* colonization and inflammation in mice ^40^. *P. acnes* CAMP factor can induce cell death of sebocytes in sebaceous glands, resulting in amplification of the inflammation response ^41^. In addition, a study reported that the *P. acnes* surface protein CAMP factor 1 stimulated keratinocytes *in vitro* by interacting directly with TLR2 ^42^.

Porphyrins are secreted by *P. acnes* and can generate reactive oxygen species that induce inflammation in keratinocytes and result in acne lesions. Johnson *et al*. showed that acne-associated *P. acnes* strains produced more porphyrins than health-associated strains isolated from individuals and that vitamin B _12_ supplementation significantly increased porphyrin production in the acne-associated strains only ^43^. Another study showed that the *P. acnes* vitamin B _12_ biosynthesis pathway was downregulated in acne patients compared with healthy individuals. Furthermore, intramuscular vitamin B _12_ supplementation repressed its own biosynthesis in *P. acnes* and promoted increased porphyrin production in healthy subjects ^44^.

Hyaluronic acid (HA) lyase is a ubiquitous enzyme with two distinct variants in the *P. acnes* population that differ in their ability to degrade HA and could be involved in the pro-inflammatory responses seen in acne. One variant is present in *P. acnes* type IA strains and is associated with acne, and the other one is in type IB and II strains and is associated mainly with soft and deep tissue infections. HA fragments interact with cell surface receptors such as CD44 and TLR2 and induce the inflammatory response ^45^.

Apart from its target activities in acne, *P. acnes* has an intriguing role in the homeostasis of the skin’s microbiome, interacting with other cutaneous microorganisms such as *Staphylococcus epidermidis*, *Streptococcus pyogenes*, and *Pseudomonas* species *.* In the microbiome of healthy skin, *S. epidermidis* may limit the overcolonization with *P. acnes* strains and reduce *P. acnes*-induced IL-6 and TNF-α production by keratinocytes. On the other hand, *P. acnes* may limit the proliferation of *S. aureus* and *S. pyogenes* by promoting triglyceride hydrolysis and propionic acid secretion. As a result, an acidic pH is maintained in the pilosebaceous follicle. A change of the microbiome composition may lead to a disturbed skin barrier and inflammation. In acne, a modified profile of *P. acnes* is noticed; different phylotypes differ between patients with and without acne ^46^. Hall *et al*. showed in cutaneous samples that when *P. acnes* was present, *Pseudomonas* species typically were not, and vice versa ^47^. Interestingly, antibiotic treatment for acne that decreases *P. acnes* colonization on the skin may also result in Gram-negative folliculitis caused by *Pseudomonas* ^48^. Megyeri *et al*. recently proposed that *P. acnes* strains may be implicated in antimicrobial defense pathways by triggering a local increase in the autophagic activity of keratinocytes ^49^.

## *P. acnes* resistance to antibiotics

The antibiotic resistance of *P. acnes* is a worldwide problem, and rates of resistance increased from 20% in 1979 to 64% in 2000; rates for tetracyclines were lower compared with rates for clindamycin and erythromycin ^50^. A study of 664 patients in the UK, Spain, Italy, Greece, Sweden, and Hungary reported that the prevalence of *P. acnes* resistance rates ranged from 50.8% to 93.6% to any antibiotic (tetracycline, macrolide, lincosamide, and streptogramin B) and that all included dermatologists who specialized in treating acne were colonized with resistant *Propionibacteria* ^51^.

A difference in the *in vitro* antibiotic susceptibility patterns of *P. acnes* among different countries is recognized ^52– 56^. A possible explanation is the fact that there are different antibiotic-prescribing habits among the countries and even different concomitant topical agents used. In studies from Korea, the UK, Colombia, Mexico, Hong Kong, Hungary, and Spain, *P. acnes* antibiotic resistance was noted in 36.7%, 55.5%, 40%, 75.5%, 54.7%, 51%, and 94% of patients with acne, respectively ( Table 2) ^57^.

**Table 2.** Different rates of *Propionibacterium acnes* antibiotic resistance in acne patients in different countries.

Study	Country, date	Patients with acne, number	Any antibiotic resistance, n (%)	Clindamycin resistance, n (%)	Erythromycin resistance, n (%)	Azithromycin resistance, n (%)	Oxytetracycline resistance, n (%)	Doxycyline resistance, n (%)	Minocycline resistance, n (%)
Moon ^59^	Korea, 2011	100 (30 *P. acnes* strains isolated)	11 (36.7)	9 (30)	8 (26.7)	NS	1 (3.3)	2 (6.7)	3 (10)
Coates ^50^	UK, 1991–2000	4,274	34.5% in 1991 55.5% in 2000	1997: ~48%	1997: 57.6%	NR	1991: 12.5% 1998: 29.9%	NR	NR
	1997	72	72 (100)	65 (90.3)	68 (94.4)	NR	38 (52.8)	NR	NR
Mendoza ^52^	Colombia, 2005, 2006	100	40%	15%	35%	NS	8%	9%	1%
Gonzalez ^53^	Northern Mexico, 2010	49	37 (75.5)	36%	46%	82%	14%	20%	0
Luk ^54^	Hong Kong, 2009	111 ( *P. acnes* isolated from 86 patients)	47 (54.7)	(53.5)	18 (20.9)	NS	14 (16.3)	14 (16.3)	14 (16.3)
Abdel-Fattah ^55^	Egypt, 2011–2012	115 ( *P. acnes* isolated from 98 patients)	NR	65 (66.3)	48 (49)	5 (5.1)	18 (18.4)	6 (6.1)	NS
Ross ^51^	1999–2001	622							
	UK		NR	50%	50%	NS	26%	NR	0
	Greece		NR	75%	75%	NS	7%	NR	0
	Hungary		51%	45%	45%	NS	0	NR	0
	Italy		NR	58%	58%	NS	0	NR	0
	Spain		94%	91%	91%	NS	5%	NR	0
	Sweden		NR	45%	45%	NS	15%	NR	0
Dumont ^56^	France, 2010	273	NR	NS	205 (75.1)	NS	26 (9.5)	26 ^a^	NS

Sampling only from closed comedones. ^a^Only the strains resistant to tetracycline (26 patients) were tested with doxycycline. NR, not reported; NS, not studied. From Dessinioti and Katsambas ^52^. Reprinted with permission from Elsevier.

Macrolide-resistant *P. acnes* is frequently isolated from patients with acne vulgaris, and the majority of resistant isolates have the 23S rRNA mutation ^58^. Long-term, low-concentration exposure to macrolides increased the resistance of *P. acnes* ^59^.

## Implications of antimicrobial resistance

The effect of acne treatments may be influenced by the presence of antibiotic‐resistant *P. acnes* ^34^. The widespread use of antibiotics to treat acne may result in the development of *P. acnes* strains with cross-resistance to different antibiotics and have possible implications in acne and other diseases where *P. acnes* may be the causative pathogen ^51^.

**Figure 2.**
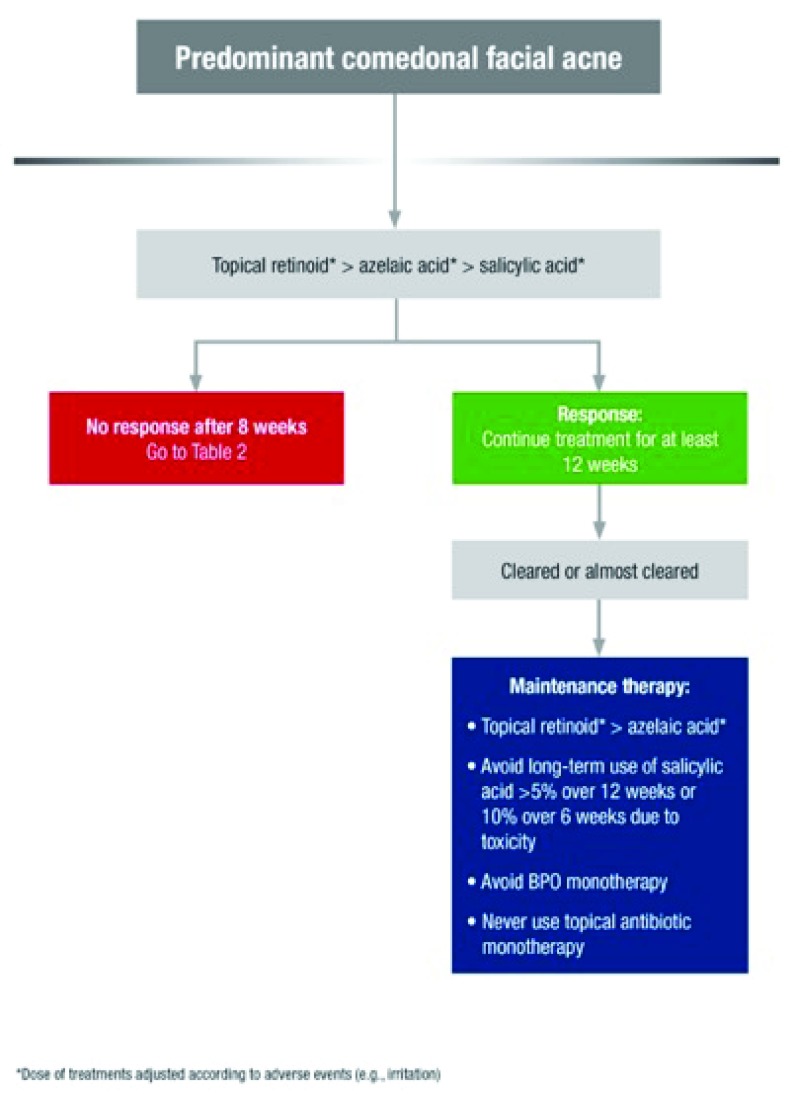
Treatment algorithm for predominant comedonal facial acne. *Dose of treatments adjusted according to adverse events (for example, irritation). BPO, benzyl peroxide. From Gollnick *et al*. ^61^. Reprinted with permission from John Wiley & Sons.

Given the frequent use of antibiotics for acne treatment, recommendations on acne treatments aim to limit the risk of antimicrobial resistance of *P. acnes* and other bacteria ^60– 62^. As a general rule, the long-term use of topical antibiotics in monotherapies should be avoided, as they may lead to an increase in antibiotic-resistant *P. acnes* ^63^. Antibiotics are not indicated for predominantly comedonal facial acne ( Figure 2). Topical antibiotics, especially as a fixed combination with benzyl peroxide (BPO) or retinoid, may be indicated for predominantly papulopustular inflammatory facial acne. Topical fixed-dose combination treatments present the advantage of a quicker onset of action and may limit the risk of antimicrobial resistance associated with antibiotic monotherapy. If the use of topical antibiotics is indicated, BPO or a topical retinoid should be added with the aim to reduce the risk of antimicrobial resistance ^64, 65^. Topical antibiotics are not suitable for maintenance acne therapy; instead, topical retinoids are preferred, and BPO is added for an antimicrobial effect if needed ^60^. BPO further exhibits antimicrobial activity against *P. acnes*. Azelaic acid inhibits the synthesis of cellular protein in aerobic and anaerobic microorganisms, such as *P. acnes*, and does not induce bacterial resistance ^61^.

**Figure 3.**
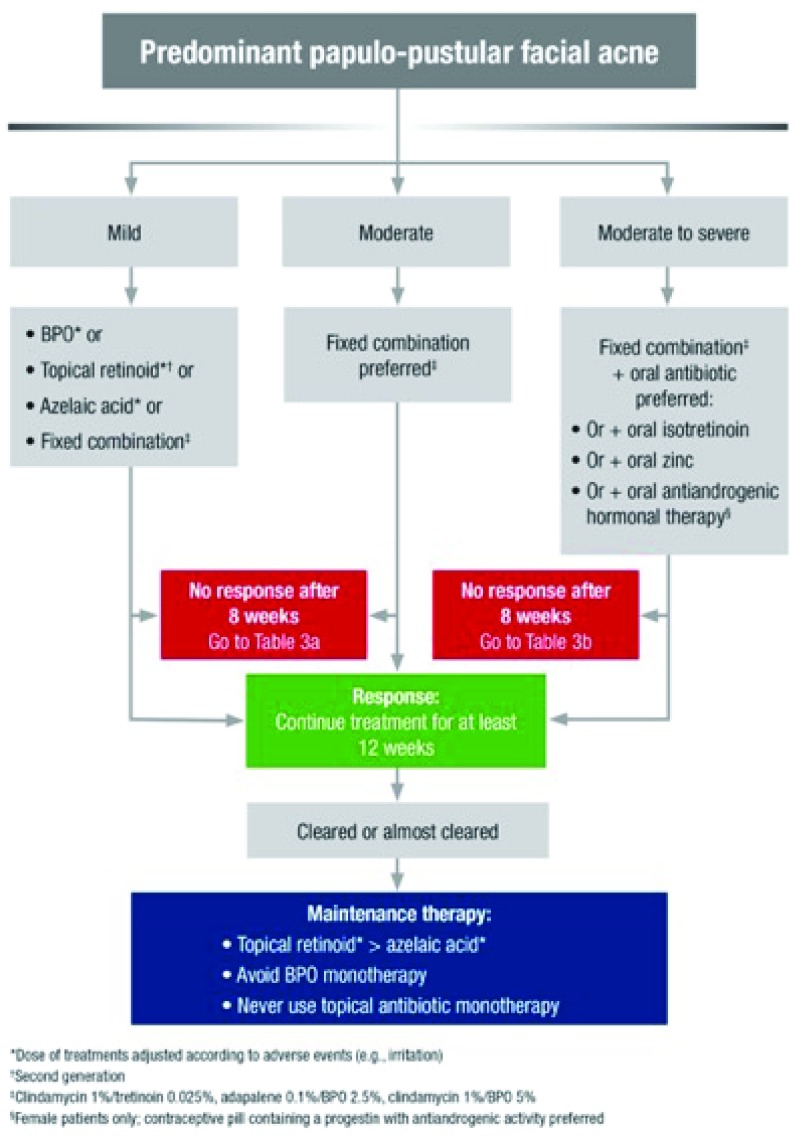
Treatment algorithm for predominant papulopustular facial acne. *Dose of treatments adjusted according to adverse events (for example, irritation). ^†^Second generation. ^‡^Clindamycin 1%/tretinoin 0.025% (not with oral antibiotic), adapalene 0.1%/BPO 2.5%, clindamycin 1%/BPO 5% (not with oral antibiotic). ^§^Female patients only; contraceptive pill containing a progestin with anti-androgenic activity preferred. BPO, benzyl peroxide. From Gollnick *et al*. ^61^. Reprinted with permission from John Wiley & Sons.

Systemic antibiotics for acne, in combination with a topical agent (BPO, retinoid, or azelaic acid), are indicated for moderate to severe inflammatory papulopustular acne and acne affecting the trunk ( Figure 3). The duration of the oral antibiotic regimen should not exceed 3 months. Oral tetracyclines (doxycycline or lymecycline) are the antibiotic of first choice for acne when a systemic antibiotic is considered ^61, 62, 66^. Treatment with oral macrolides should be avoided because of high rates of antimicrobial resistance reported for *P. acnes* worldwide ^51^.

A study of 56 patients with cystic or severe acne vulgaris treated with oral isotretinoin (1 mg/kg per day) reported that the colonization of the skin with *P. acnes* was modified; oral isotretinoin, though not an antibiotic, correlated with a reduction in the numbers of *P. acnes*, including isolates resistant to antibiotics, that were cultured from the cheeks, but there was no effect in *P. acnes* sampled from other anatomic sites ^67^.

Emerging off-label therapeutic modalities for acne, such as topical photodynamic therapy (PDT) with photoactivation of aminolaevulinic acid (ALA) or methyl aminolaevulinic acid (MAL), target P *. acnes*, underlying its role in the pathogenesis of acne ^68^. The mode of action of PDT includes not only photodynamic damage of the sebaceous gland but also the photodestruction of *P. acnes* ^69, 70^.

The potential for vaccination against *P. acnes* was investigated, and relevant studies initially stopped in 2011, as effectiveness in humans with acne was not shown ^71^. Interestingly, a recent study reported the efficacy of CAMP factor antibodies in the neutralization of the acne inflammatory response in *ex vivo* acne models; the incubation of *ex vivo* acne skin explants from acne patients with monoclonal antibodies (mAbs) to the *P. acnes*-secreted CAMP factor diminished the amounts of pro-inflammatory cytokines IL-8 and IL-1β ^40^. The authors also proposed that the injection of the mAb to CAMP factor directly into acne lesions may prove to be beneficial ^40^.

## Conclusions

Significant progress has been made in understanding the role of *P. acnes* in the pathogenesis of acne. Although there is no quantitative difference of *P. acnes* among patients with acne and healthy individuals, *P. acnes* phylogenic groups may display distinct genetic and phenotypic characteristics. Different ** phylotypes may induce distinct immune responses, and the *P. acnes* biofilm has been reported more frequently in patients with acne. Furthermore, *P. acnes* plays important roles in the homeostasis of the skin’s microbiome, interacting with other cutaneous microorganisms such as *S. epidermidis*, *S. pyogenes*, and *Pseudomonas* species. Non-antibiotic approaches targeting *P. acnes* without inducing antibiotic resistance may improve patient outcomes in acne while avoiding public health issues.
